# How gender- and violence-related norms affect self-esteem among adolescent refugee girls living in Ethiopia

**DOI:** 10.1017/gmh.2017.28

**Published:** 2018-01-17

**Authors:** L. Stark, K. Asghar, I. Seff, B. Cislaghi, G. Yu, T. Tesfay Gessesse, J. Eoomkham, A. Assazenew Baysa, K. Falb

**Affiliations:** 1 Department of Population and Family Health, Columbia University Mailman School of Public Health, 60 Haven Ave B-4 Suite 432, New York, NY, USA; 2 Department of Global Health and Development, London School of Hygiene and Tropical Medicine, Keppel Street, London, UK; 3 New York University College of Nursing, 433 First Avenue, New York, NY, USA; 4 Independent Consultant, Mekele, Ethiopia; 5 The International Rescue Committee, TK International Bldg. 6th Floor, Bole Rd, Addis Ababa, Ethiopia; 6 The International Rescue Committee, 1730M St NW, Suite 505, Washington DC, USA

**Keywords:** Adolescent health, conflict, gender norms, resilience, self-esteem

## Abstract

**Background.:**

Evidence suggests adolescent self-esteem is influenced by beliefs of how individuals in their reference group perceive them. However, few studies examine how gender- and violence-related social norms affect self-esteem among refugee populations. This paper explores relationships between gender inequitable and victim-blaming social norms, personal attitudes, and self-esteem among adolescent girls participating in a life skills program in three Ethiopian refugee camps.

**Methods.:**

Ordinary least squares multivariable regression analysis was used to assess the associations between attitudes and social norms, and self-esteem. Key independent variables of interest included a scale measuring personal attitudes toward gender inequitable norms, a measure of perceived injunctive norms capturing how a girl believed her family and community would react if she was raped, and a peer-group measure of collective descriptive norms surrounding gender inequity. The key outcome variable, self-esteem, was measured using the Rosenberg self-esteem scale.

**Results.:**

Girl's personal attitudes toward gender inequitable norms were not significantly predictive of self-esteem at endline, when adjusting for other covariates. Collective peer norms surrounding the same gender inequitable statements were significantly predictive of self-esteem at endline (*ß* = −0.130; *p*  =  0.024). Additionally, perceived injunctive norms surrounding family and community-based sanctions for victims of forced sex were associated with a decline in self-esteem at endline (*ß* = −0.103; *p*  =  0.014). Significant findings for collective descriptive norms and injunctive norms remained when controlling for all three constructs simultaneously.

**Conclusions.:**

Findings suggest shifting collective norms around gender inequity, particularly at the community and peer levels, may sustainably support the safety and well-being of adolescent girls in refugee settings.

## Introduction

Self-esteem, defined as a favorable or unfavorable attitude toward the self, is associated with physical and mental well-being in young adulthood, even when controlling for factors such as depression, gender, and socio-economic status (Rosenberg, 1965; Swallow & Kuiper, 1988; Trzesniewski *et al.* 2006; Khosravi *et al.* 2016). Although self-esteem results from an internal process of evaluating one's own skills and competencies, that process is influenced by what adolescents believe parents and peers think of them (Harter, 1993). In fact, many early self-esteem theorists have posited that self-esteem is a predominantly social construct, and an individual's concept of self is significantly shaped by how he or she believes they are perceived by others in a given reference group (Cooley, 1902).

Social norms theory, which is primarily used to explain how an individual's perceptions of others’ behaviors and opinions shapes his or her own behavior, can help elucidate how the phenomenon described above may be operationalized. Social norms are the unwritten rules regulating what is appropriate in a society or group. There exist two primary schools of thought related to social norms: the first suggesting that norms are regularities in people's behavior or attitudes, the second asserting that norms are beliefs about what others do and approve of (Knight Lapinski & Rimal, 2005; Morris *et al.* 2015). Scholars in the first stream of thought often refer to norms as the collective behaviors and attitudes shared by a society; what Chung & Rimal (2016) call ‘collective norms’. Scholars in the second stream of thought instead think of norms as beliefs (right or wrong) about other people's attitudes and behaviors. Seminal work by social psychologist Cialdini distinguished between two types of social norms as beliefs: descriptive norms, the belief one has about what others commonly do, and injunctive norms, the belief one has about what others approve and disapprove of (Cialdini *et al.* 2006). Importantly, norms have a stronger effect when they are salient, that is, when situational cues activate them (Cialdini *et al.* 1991; Cialdini & Goldstein, 2004; Cislaghi & Heise, under review). People often comply with social norms as they anticipate social rewards for doing so and social punishment if they do otherwise (Elsenbroich & Gilbert, 2014). Social norms are considered distinct from personal attitudes. Although personal attitudes may be influenced by social norms, they represent an individual's own evaluation of a belief or behavior with some level of approval or disapproval (Mackie *et al.* 2015; Young, 2015; Chung & Rimal, 2016; Miller & Prentice, 2016).

Existing research demonstrates complex relationships between personal attitudes, different types of norms, and well-being. For example, a 20-country study found that self-esteem was associated with collective norms rather than personal attitudes (Becker *et al.* 2014). Differences in these relationships may be attributed to different pathways through which messaging about norms may be activated in the brain (Morris *et al.* 2015). Adolescents may be especially sensitive to social norms, since receptiveness to social stimuli peaks during adolescence, and adolescents may perceive greater rewards for conforming (Dijkstra & Cillessen, 2013; Berger & Caravita, 2016). While protective and prosocial norms can positively impact adolescents’ well-being (Viner *et al.* 2012), empirical evidence has shown that social norms can also have a harmful influence on adolescents’ well-being, increasing their likelihood of engaging in behaviors such as smoking (Eisenberg & Forster, 2003), drinking alcohol (Dams-O'Connor *et al.* 2007), and using recreational drugs (Jiloha, 2009).

Research from high-income countries shows that girls exhibit significantly lower self-esteem than their male counterparts as early as adolescence (Allgood-Merten & Stockard, 1991). While this lower self-esteem may in part be related to adolescent girls’ greater risk of depression as compared with boys’ (Cohen *et al.* 1993), some empirical evidence suggests that women and girls who internalize gender norms that devalue women often hold themselves in lower esteem (Katz *et al.* 2002).

Despite the widely recognized effect that norms have on people's health and well-being (Chung & Rimal, 2016; Cislaghi & Heise, under review), there is a dearth of evidence on how gender- and violence-related social norms affect self-esteem, especially among refugee populations. Yet, understanding these complex relationships is particularly critical for these vulnerable populations as they often face greater risks of gender-based marginalization and violence. Nascent evidence from refugee settings indicates that individuals’ acceptance of intimate partner violence (IPV) and violence victimization may jointly be associated with lower levels of hope, and that these associations may not be consistent across different forms of violence (Stark *et al.* 2017*a*, *b*). Additionally, adherence to gender inequitable attitudes may be stronger among women, and women may reinforce such norms when in the presence of same-sex peers (Gage & Hutchinson, 2006; Uthman *et al.* 2009; Trott *et al.* 2016).

The study presented in this article explores these dynamic relationships among female adolescents from Sudan and South Sudan currently living in Ethiopian refugee camps. As of 2013, there were more than 500 000 refugees living in Ethiopia, of whom 135 926 were South Sudanese and 32 520 were Sudanese (UNHCR Operation in Ethiopia 2014). Adolescent refugees in this setting have been found to exhibit elevated rates of mental health distress, including depression, anxiety, and post-traumatic stress (Tempany, 2009). These mental health issues may result from exposure to conflict-related trauma in their home country, the hardships associated with forced migration, or the stressors of living in a foreign environment without access to one's normal support system (Tempany, 2009). Additionally, while gender norms and attitudes data among this population are limited in the literature, one study found that 82% of women and 81% of men in different areas of South Sudan agreed that ‘a woman should tolerate violence in order to keep her family together’; 68% of women and 63% of men agreed that ‘there are times when a woman deserves to be beaten’; and 56% of both men and women believed that women should not disclose physical violence committed by husbands to others (Scott, 2013). This article explores the relationship between gender inequitable and victim-blaming social norms, personal attitudes, and self-esteem among a sample of adolescent refugee girls participating in a life skills program in three refugee camps in Ethiopia.

## Methods

### Study setting, participants, and procedures

This study took place in three refugee camps in the Benishangul-Gumuz regional state in Ethiopia: Sherkole, Tongo, and Bambasi. The camps are home to tens of thousands of refugees, predominantly from Sudan and South Sudan (Falb *et al.* 2016). Data for this analysis are drawn from the Creating Opportunities through Mentorship, Parental Involvement, and Safe Spaces (COMPASS) wait-list cluster-randomized controlled trial (for further details of the trial, see Falb *et al.*). Study inclusion criteria included: (1) being a girl 13–19 years of age; (2) residing in Sherkole, Tongo, and Bambasi refugee camps; and (3) possessing verbal proficiency in one of the four primary languages spoken within the camps (Funj, Regareg, Ingessena Kulelek, and Maban). Prior to the baseline study, IRC's research and program teams conducted language assessments and identified 19 tribal languages in the study camps, none of which are written. Verbal proficiency in one of the four most frequently spoken languages was assessed using a series of questions about girls’ typical language use at home, with friends, and at the market. For ethical reasons, girls with significant cognitive impairments or physical disabilities that would prevent independent completion of the questionnaire were not eligible for inclusion.

Caregivers, married girls, and girls over age 18 provided direct verbal consent, while verbal assent was obtained for all unmarried girls ages 13–17. Consent and assent were obtained in confidential spaces. Data were collected at two time points (July–September 2015 and July–September 2016). All surveys were self-administered using Audio Computer-Assisted Self-Interview (ACASI) programming in a private setting (Falb *et al.* 2017).

All study protocols were approved by the Institutional Review Board of the Columbia University Medical Center (Protocol #AAAP6855), the Administration for Refugee and Returnee Affairs in Ethiopia, and the International Rescue Committee's internal review board (Protocol #WPE 1.00.003).

### Measures

#### Independent variables

Our analysis examines the relationships between self-esteem and three primary constructs: personal attitudes regarding gender role dynamics, injunctive norms regarding sexual violence and collective peer norms toward gender role dynamics. Our measure of personal attitudes surrounding gender role dynamics was constructed using four survey questions. Girls were asked to indicate their level of agreement with each of the following four statements: ‘*It is a female's responsibility to avoid getting pregnant*’; ‘*A male should have the final word about decisions in his home’;* ‘*A female should tolerate violence to keep the family* together’; and ‘*It is okay for a male to beat his wife if she will not have sex with him*’. For each statement, respondents were assigned a ‘1’ if they agreed and a ‘0’ if they disagreed. The final measure was created by summing the values of these four items, such that a value of ‘4’ reflected highly gender inequitable norms and a value of ‘0’ indicated gender equitable norms.

Our measure of perceived injunctive norms reflected how a girl believed her family and community would react if she was raped. Perceived injunctive norms are described as what one individual thinks others approve and disapprove, but investigating people's reactions to a given action have been found to be satisfactory proxy measures (Cislaghi & Heise, 2017). Girls were asked to indicate their level of binary agreement with two sanction-related statements: ‘*My family would blame me if I was forced to have sex*’ and ‘*My community would force me to marry a man if he forced me to have sex*’. Girls were assigned a ‘1’ if they agreed with the statement, and ‘0’ if they disagreed. The resulting measure was the sum of these two values; the final composite score could take a value of 0, 1, or 2, where 2 indicated greater perceptions of victim blaming norms.

Finally, we used a measure of collective peer norms toward gender inequity (Mackie *et al.* 2015). To measure collective norms, we aggregated individuals’ attitudes at the zonal level (geographic areas within the camps) to create a measure of what was considered ‘normal’ in a given reference group. This measure reflects the mean value of personal attitudes toward gender inequity described above, among all sample girls in a given zone. The study sample included respondents from 12 zones. Utilization of this variable allowed us to examine how gender inequitable norms might differentially impact a girl's well-being at the individual *v*. community or peer level. Other individual-level variables in our analysis included treatment status (1  =  intervention; 0  =  control), age in years, living with an intimate partner at endline (1  =  yes; 0  =  no), and whether or not a girl attended school between baseline and endline (1  =  yes; 0  =  no).

#### Outcome of interest

Our primary outcome of interest, self-esteem, was measured using the Rosenberg self-esteem scale (RSES). The RSES was designed to capture adolescents’ sense of self-worth by assessing both positive and negative feelings about themselves (Rosenberg, 1965). The RSES has been shown to be correlated with external personality traits as well as mental health conditions, including depression and anxiety (Rosenberg, 1979; Schmitt & Allik, 2005). Respondents are presented with 10 statements and asked to indicate their level of agreement using a four-point Likert scale, ranging from ‘strongly agree’ to ‘strongly disagree’. Each item may take an ordinal value from 1 to 4, with 4 indicating strong agreement. The composite score is created by summing the values assigned to each of the 10 items, with some items being reverse-coded. The final scale may take a value from 10 to 40, where a higher value indicates greater self-esteem.

The RSES was first developed in 1965 and administered to high school students from 10 randomly selected New York high schools (Rosenberg, 1965). In subsequent years, the scale has been adapted for a variety of groups. The RSES exhibits high internal consistency, with a Guttman scale coefficient of reducibility at 0.92, as well as demonstrated test–retest reliability and stability (Rosenberg, 1979). Schmitt & Allik (2005) examined cross-cultural appropriateness of the scale in 28 languages and 53 countries, and determined the scale was generally valid for both individualist and collectivist societies. Previous use of the RSES in Ethiopia has demonstrated sufficient reliability, with a Cronbach's *α* of 0.64 (Schmitt & Allik, 2005).

Question items for the RSES were administered as part of the survey using ACASI. Four of the five reverse-coded items from the original RSES were modified for use in this study. Findings from our formative research and pilot testing in the study population revealed that some of the negatively phrased items were confusing to girls. For this reason, items such as ‘I feel I don't have much to be proud of’ were rephrased as ‘I feel I have much to be proud of’; the final scale contains only one item that is reverse-coded. The Cronbach's *α* for this scale in this study was 0.86 at baseline and 0.88 at endline, demonstrating satisfactory reliability.

### Statistical analysis

The sample size for this study was calculated assuming statistical power of 80% and a two-sided *α* of 0.05 to detect a 20% decline in the primary outcome of the COMPASS study (the incidence of past-year sexual violence) in the intervention arm as compared with the waitlist arm. Calculations estimated approximately 12–15 girls in each cluster and assumed an intraclass correlation coefficient for the study's primary outcome of 0.06 to account for clustering. Final calculations required 62 clusters, with 31 groups in each treatment arm, and at least 896 girls powered at the primary outcome of sexual violence. Self-esteem was subsequently examined in this sample as a secondary outcome of interest.

Linear mixed regression analyses were used to assess the relationships between attitudes and social norms, and self-esteem. Model 1 examines the relationship between individual attitudes toward gender inequitable norms at baseline and self-esteem at endline. Model 2 estimates the *β* coefficient between individual-level perceived injunctive norms at baseline and self-esteem at endline. In model 3, we assess the extent to which a ‘peer group effect’ influences a girl's self-esteem. Finally, a fourth model examines the impact of all three constructs simultaneously on the outcome of interest. Multicollinearity of this fourth model was assessed using a variance inflation factor (VIF) and a predetermined threshold of 2.5. All models control for treatment status, baseline self-esteem, age in years, whether a girl lives with an intimate partner, and whether she has attended school between baseline and endline. Standard errors are clustered at the level of randomization, which also served as the peer group assignment for COMPASS program sessions.

Due to attrition between baseline and follow-up surveys and item non-response for many sensitive questions, predictors were missing for approximately 10–20% of girls, and at least one item in the endline Rosenberg scale was missing for approximately 35% of girls. *T* tests were used to assess whether missingness of the outcome was associated with predictors. We conducted a sensitivity analysis by carrying out our main set of analyses on imputed data. A multiple imputation approach was used, generating a set of five imputations in Stata with ‘mi impute’. Thus, the final average values reflect a reasonable estimate of a full dataset. All analyses were implemented using Stata 14.

## Results

The full baseline sample included 919 girls (Table 1). The average age of study participants at baseline was 14.59 years (s.d. 1.51). Funj was the most common language spoken among participants. Twenty-five percent of girls in the sample report living with an intimate partner at baseline, and no statistically significant differences were observed between treatment and control arms. On average, respondents had completed 3.99 years of school and 58% of respondents had attended school in the 12 months preceding baseline data collection.

**Table 1.** Baseline characteristics

	Total (s.d.)	Treatment (s.d.)	Control (s.d.)	*p* value
Age (years)	14.60 (1.51)	14.58 (1.52)	14.62 (1.51)	0.723
Language				<0.001
Ingessena Kulelek	0.10 (0.30)	0.11 (0.32)	0.09 (0.29)	
Funj	0.66 (0.47)	0.72 (0.45)	0.61 (0.49)	
Maban	0.17 (0.37)	0.15 (0.36)	0.19 (0.39)	
Regareg	0.07 (0.25)	0.02 (0.15)	0.12 (0.32)	
Camp				0.003
Bambasi	0.37 (0.48)	0.43 (0.50)	0.31 (0.46)	
Tongo	0.29 (0.45)	0.26 (0.44)	0.32 (0.47)	
Sherkole	0.35 (0.48)	0.32 (0.47)	0.37 (0.49)	
Parents in household				0.014
Live with father only	0.20 (0.40)	0.18 (0.39)	0.21 (0.41)	
Live with mother only	0.27 (0.45)	0.31 (0.46)	0.24 (0.43)	
Live with both parents	0.45 (0.50)	0.45 (0.50)	0.45 (0.50)	
Live with neither parent	0.08 (0.27)	0.06 (0.24)	0.10 (0.31)	
Live with an intimate partner	0.25 (0.43)	0.23 (0.42)	0.26 (0.44)	0.289
Attended school in the last 12 months	0.58 (0.49)	0.60 (0.49)	0.56 (0.50)	0.231
Years of schooling (years)	3.99 (3.85)	4.02 (4.02)	3.97 (3.67)	0.861
Individual attitudes, gender inequitable norms	2.03 (1.35)	2.06 (1.34)	1.99 (1.37)	0.495
Perceived injunctive norms	0.52 (0.67)	0.56 (0.68)	0.47 (0.65)	0.056
Rosenberg self-esteem	31.03 (5.61)	31.29 (5.26)	30.77 (5.92)	0.233

Note: all values reflect proportions in the sample, except when specified.

Missingness of the outcome of interest, Rosenberg self-esteem at endline, was found to be associated with a girl's individual attitudes toward gender equitable norms. Specifically, girls with greater gender equitable attitudes at baseline were more likely to have a missing value for at least one item in the RSES at endline. A girl's perceived injunctive norms variable was not associated with missingness of the outcome of interest.

A girl's personal gender inequitable attitudes were not significantly predictive of self-esteem at endline (*p*  =  0.836) (Table 2). In other words, the number of statements supporting gender inequity that a girl agreed with was not associated with having a higher or lower level of self-esteem. In contrast, one standard deviation increase in the perceived injunctive norms scale was associated with a 0.103 standard deviation decline in self-esteem at endline (*p*  =  0.014).

**Table 2.** Estimates of Rosenberg self-esteem scale at endline, multilevel regressions

	(1)		(2)		(3)		(4)	
	*β* (*p* value)	(95% CI)	*β* (*p* value)	(95% CI)	*β* (*p* value)	(95% CI)	*β* (*p* value)	(95% CI)
Self-esteem at baseline	0.038*** (0.000)	(0.02–0.06)	0.042*** (0.000)	(0.03–0.06)	0.042*** (0.000)	(0.03–0.06)	0.032** (0.001)	(0.01–0.05)
Intervention	0.077 (0.471)	(−0.14 to 0.29)	0.075 (0.456)	(−0.12 to 0.27)	0.057 (0.561)	(−0.14 to 0.25)	0.107 (0.257)	(−0.08 to 0.29)
Individual attitudes, gender inequitable norms	−0.000 (0.836)	(−0.11 to 0.09)					0.102 (0.052)	(−0.00 to 0.20)
Perceived injunctive norms			−0.103* (0.014)	(−0.18 to −0.02)			−0.122** (0.003)	(−0.20 to −0.04)
Collective peer attitudes, gender inequitable norms					−0.130* (0.024)	(−0.24 to −0.02)	−0.128* (0.042)	(−0.25 to −0.00)
*R*^2^	0.268		0.286		0.287		0.299	
AIC	1148.03		1177.18		1245.29		1077.10	
*N*	444		459		484		435	

Note: coefficients are standardized. Standard errors are clustered at the group level of randomization. Coefficients are statistically significant at: *p  <  0.05, **p  <  0.01, ***p  <  0.001. All models control for age in years at baseline, whether a girl has attended school between baseline and endline, living with an intimate partner at endline, and self-esteem at baseline. The mean VIF for model 4 is 1.2 and the VIF for any single predictor does not exceed 1.6.

The results from model 3 demonstrate that collective peer norms were also significantly predictive of self-esteem at endline; one standard deviation increase in a girl's collective peer norms scale resulted in a decline in her Rosenberg self-esteem score of 0.130 standard deviations (*p*  =  0.024). The contrasting results from models 1 and 3 suggest that, although personal norms toward gender inequity did not impact self-esteem, living within a peer group that promoted greater gender inequity had significantly negative effects on girls’ self-esteem.

The findings from models 1 through 3 hold when examining the effects of these three factors on self-esteem simultaneously (model 4). One standard deviation increase in perceived injunctive norms and collective peer norms was associated with 0.122 and 0.128 standard deviation declines in the RSES, respectively, when controlling for all covariates.

The findings from models 1 through 3 were robust to our sensitivity analyses. We observed only one different result when running model 4 on the imputed data; while perceived injunctive norms and collective peer attitudes were still negatively associated with self-esteem, we found that individual attitudes toward gender inequitable norms were positively associated with self-esteem. This suggests our primary analysis, that individual attitudes are not associated with self-esteem, may be slightly less robust than our other findings.

## Discussion

Low adolescent self-esteem has been associated with poor physical and mental well-being in young adulthood. Previous studies have demonstrated a bi-directional relationship between low self-esteem and depression and hopelessness, and there exists strong evidence linking low self-esteem to suicide ideation (Harter, 1993). These relationships remain significant even when controlling for other factors such as gender and socio-economic status (Trzesniewski *et al.* 2006). Further, research suggests self-esteem can influence women's and girls’ likelihood of violence victimization, a risk that is already elevated in humanitarian settings (Marsh *et al.* 2006). A study examining risk factors for experiencing gender-based violence (GBV) post-hurricane Katrina found that women with low self-esteem had 2.3 greater odds of exposure to GBV than did women with high self-esteem (Anastario *et al.* 2008).

Our study finds that, while a girl's personal attitudes supporting gender inequitable roles have no effect on her self-esteem, the collective attitudes of her peers (what we called collective peer norms) toward the same items are significantly associated with lower self-esteem. In other words, a girl's agreement with gender inequitable statements did not translate to a diminished concept of self; in contrast, being surrounded by peers who adhere to norms that devalue girls resulted in lower self-esteem. Collective peer norms are thus powerful predictors of well-being among refugee adolescent girls in our study. Perceived injunctive norms that support victim-blaming in the event of forced sex were also predictive of lower self-esteem among these adolescent girls. A girl who believed she would be blamed by her family and community and receive minimal social support if forced to have sex exhibited lower self-esteem. Recall that perceived injunctive norms have a stronger effect when detectable social cues make them salient. Given the salience of violence in refugee settlements, it is not surprising that perceived injunctive norms regarding sanctions for survivors of violence would influence a girl's self-esteem, even if she has not herself been a victim of sexual violence.

This study, along with existing theory and evidence, hints at a potential ‘herd immunity’^†^^1^ effect, where equitable gender- and violence-related norms among a group may be protective of well-being, and inequitable norms may exacerbate poor mental health. This herd effect is already a foundational assumption of programs that utilize social norms theory to induce behavior change. While targeting personal attitudes is helpful, influencing broader societal norms is critical (Mackie *et al.* 2015; Cislaghi *et al.* 2016). From a programmatic perspective, these findings point to the need for more community-based interventions to leverage broader social norms change. Such an approach has the added benefit of potentially benefitting marginalized individuals who might not otherwise participate in these interventions. Although these individuals may not receive program messaging directly, norm changes at the community level may still positively affect their mental well-being.

Additionally, previous research demonstrates that adolescents are particularly susceptible to peer influence, more so than at other stages in the life course (Halfon & Hochstein, 2002; Steinberg, 2005; Albert *et al.* 2013). In addition to shaping the health and behaviors of adolescents, the peer group can have a profound impact on the construction of identity during this phase in the life course (Zarrett & Eccles, 2006). As adolescents develop their sense of identity, they begin to self-categorize themselves as belonging to certain groups; for example, adolescent girls identifying as ‘female’ will internalize the characteristics attributed to females among their peer group (Harris, 1995). Following this logic, a girl whose peers devalue women and girls may hold herself in lower esteem as well, even if she does not subscribe to the same gender inequitable attitudes. Consequently, community social norms programs that intend to reach adolescents would be wise to include a component that specifically targets peer norms.

Further, because self-esteem has been shown to be highly correlated with self-efficacy among adolescent girls, the salience and internalization of gender inequitable norms may affect girls’ behaviors as they relate to GBV risks and responses (Allgood-Merten & Stockard, 1991). This finding is especially relevant for refugee women and adolescent girls, who face increased risks of experiencing violence during, and after conflict (Hossain *et al.* 2014). Previous studies show that adolescent acceptance of norms condoning traditional gender roles and IPV is strongly associated with later violence exposure (Lichter & McCloskey, 2004). Our findings suggest that targeting changes in gender inequitable norms at the community level may strengthen girls’ self-esteem and resilience in the event of sexual violence and plausibly reduce the risk of victimization as well.

As we were experimenting with norms measurement, one limitation of this study may stem from our choice to use zones as the geographic unit when creating our measure of collective peer norms. This decision was based on three primary criteria. First, zones comprise individuals living within close proximity of each other, ensuring girls within a zone come into regular contact with one another. Second, the sample size of girls within each zone was sufficiently large to accurately represent ‘collective peer norms’. Third, the sample size of zones was large enough to capture variation in the measure of peer norms across zones. However, it is possible that other levels of aggregation would serve as more appropriate proxy reference groups for adolescent girls. For example, using camps rather than zones would minimize contamination across clusters, though it would also minimize cluster sample size and variation. Additionally, while the girls in our sample spoke informal Arabic as a means to communicate across different ethnicities in markets, schools, and other public spaces within zones, girls may more closely identify with others who share their ethnicity and language. Finally, because this study was funded to assess the impact of the intervention on the primary outcome of sexual violence, it was not powered specifically for detecting changes in self-esteem; the final number of clusters and girls in this sample may need to be increased in future studies evaluating similar relationships. Future research might consider carrying out similar analyses with different types of reference groups and with people of different gender and age.

## Conclusion

The positive benefits of self-esteem are well established, and may have profound importance for a young generation emerging from conflict and displacement. Despite the destruction and disruption that conflict brings, unique opportunities may emerge to shift social norms related to gender and violence. These norm shifts may, in turn, have important and long-lasting effects on the well-being and safety of women and girls. Our research offers insight into pathways for strengthening self-esteem among adolescent girls, and highlights the importance of current efforts to shift social norms in humanitarian settings.

**Clinical Trials Registration:** NCT02384642

## Footnotes

† The notes appear after the main text.

## Note

1 Herd immunity derives from infectious disease public health, and is used to describe the phenomenon wherein an individual who is not immune to an infectious disease is still protected from infection because a large percentage of the population has become immune to an infection.

## References

[ref44] Albert D, Chein J, Steinberg L (2013). Peer Influences on Adolescent Decision Making. Current directions in psychological science 22, 114–120. doi:10.1177/0963721412471347 25544805 10.1177/0963721412471347 PMC4276317

[ref45] Anastario MP, Larrance R, Lawry L (2008). Using mental health indicators to identify postdisaster gender-based violence among women displaced by Hurricane Katrina. J Womens Health (Larchmt) 17, 1437–1444. doi:10.1089/jwh.2007.0694 18945206 10.1089/jwh.2007.0694

[ref1] Allgood-Merten B, Stockard J (1991). Sex role identity and self-esteem: a comparison of children and adolescents. Sex Roles 25, 129–139.

[ref2] Becker M, Vignoles VL, Owe E, Easterbrook MJ, Brown R, Smith PB, Bond MH, Regalia C, Manzi C, Brambilla M, Aldhafri S, Gonzalez R, Carrasco D, Paz Cadena M, Lay S, Schweiger Gallo I, Torres A, Camino L, Ozgen E, Guner UE, Yamakoglu N, Silveira Lemos FC, Trujillo EV, Balanta P, Macapagal ME, Cristina Ferreira M, Herman G, De Sauvage I, Bourguignon D, Wang Q, Fulop M, Harb C, Chybicka A, Mekonnen KH, Martin M, Nizharadze G, Gavreliuc A, Buitendach J, Valk A, Koller SH (2014). Cultural bases for self-evaluation: seeing oneself positively in different cultural contexts. Personality and Social Psychology Bulletin 40, 657–675. 24523298 10.1177/0146167214522836

[ref3] Berger C, Caravita SC (2016). Why do early adolescents bully? Exploring the influence of prestige norms on social and psychological motives to bully. Journal of Adolescence 46, 45–56. 26584218 10.1016/j.adolescence.2015.10.020

[ref4] Chung A, Rimal RN (2016). Social norms: a review. Review of Communication Research 2016, 1–28.

[ref5] Cialdini RB, Demaine LJ, Sagarin BJ, Barret DW, Rhoads K, Winter PL (2006). Managing social norms for persuasive impact. Social Influence 1, 3–15.

[ref6] Cialdini RB, Goldstein NJ (2004). Social influence: compliance and conformity. Annual Review of Psychology 55, 591–621. 10.1146/annurev.psych.55.090902.142015 14744228

[ref7] Cialdini RB, Kallgren CA, Reno RR (1991). A focus theory of normative conduct: a theoretical refinement and reevaluation of the role of norms in human behavior. Advances in Experimental Social Psychology 24, 1–243.

[ref8] Cislaghi B, Gillespie D, Mackie G (2016). Values Deliberation and Collective Action: Community Empowerment in Rural Senegal. Palgrave MacMillan: New York.

[ref9] Cislaghi B, Heise L (2017). Measuring Gender-Related Social Norms: Report of a Meeting. Baltimore Maryland, June 14–15, 2016. LSHTM: London.

[ref10] Cislaghi B, Heise L (under review). Four avenues of normative influence. 10.1037/hea0000618 29672098

[ref11] Cohen P, Cohen J, Kasen S, Velez CN, Hartmark C, Johnson J, Rojas M, Brook J, Streuning EL (1993). An epidemiological study of disorders in late childhood and adolescence – I. Age- and gender-specific prevalence. Journal of Child Psychology and Psychiatry 34, 851–867. 8408371 10.1111/j.1469-7610.1993.tb01094.x

[ref12] Cooley C (1902). Human Nature and the Social Order. Scribner's Sons: New York.

[ref13] Dams-O'Connor K, Martin JL, Martens MP (2007). Social norms and alcohol consumption among intercollegiate athletes: the role of athlete and nonathlete reference groups. Addictive Behavior 32, 2657–2666. 10.1016/j.addbeh.2007.04.030 17544589

[ref14] Dijkstra JK, Cillessen AHN (2013). Popularity and adolescent friendship networks: selection and influence dynamics. Developmental Psychology 49, 1242–1252. 22985296 10.1037/a0030098

[ref15] Eisenberg ME, Forster JL (2003). Adolescent smoking behavior. American Journal of Preventive Medicine 25, 122–128. 10.1016/s0749-3797(03)00116-8 12880879

[ref16] Elsenbroich C, Gilbert N (2014). Theorising norms In Modelling Norms (ed. C. Elsenbroich and N. Gilbert). Springer Netherlands: Amsterdam, 143–149

[ref17] Falb K, Tanner S, Asghar K, Souidi S, Mierzwa S, Assazenew A, Bakomere T, Mallinga P, Robinette K, Tibebu W, Stark L (2017). Implementation of Audio-Computer Assisted Self-Interview (ACASI) among adolescent girls in humanitarian settings: feasibility, acceptability, and lessons learned. Conflict and Health 10, 32. 28053657 10.1186/s13031-016-0098-1 PMC5209867

[ref18] Falb KL, Tanner S, Ward L, Erksine D, Noble E, Assazenew A, Bakomere T, Graybill E, Lowry C, Mallinga P, Neiman A, Poulton C, Robinette K, Sommer M, Stark L (2016). Creating opportunities through mentorship, parental involvement, and safe spaces (COMPASS) program: multi-country study protocol to protect girls from violence in humanitarian settings. BMC Public Health 16, 231. 26945586 10.1186/s12889-016-2894-3 PMC4779562

[ref19] Gage AJ, Hutchinson PL (2006). Power, control, and intimate partner sexual violence in Haiti. Archives of Sexual Behavior 35, 11–24. 16502150 10.1007/s10508-006-8991-0

[ref20] Halfon N, Hochstein M (2002). Life course health development: an integrated framework for developing health, policy, and research. The Milbank Quarterly 80, 433–479. Retrieved from http://www.jstor.org/stable/3350561. 12233246 10.1111/1468-0009.00019 PMC2690118

[ref21] Harris JR (1995). Where is the child's environment? A group socialization theory of development. Psychological Review 102, 458–489.

[ref22] Harter S (1993). Causes and consequences of low self-esteem in children and adolescents In Self-Esteem: The Puzzle of Low Self-Regard (ed. RF Baumeister). Springer US: Boston, MA, 87–116.

[ref46] Hossain M, Zimmerman C, Watts C (2014). Preventing violence against women and girls in conflict. Lancet 383, 2021–2022. doi:10.1016/s0140-6736(14)60964-8 24923526 10.1016/S0140-6736(14)60964-8

[ref23] Jiloha RC (2009). Social and cultural aspects of drug abuse in adolescents. Delhi Psychiatric Journal 12, 167–175.

[ref24] Katz J, Joiner TE, Kwon P (2002). Membership in a devalued social group and emotional well-being: developing a model of personal self-esteem, collective self-esteem, and group socialization. Sex Roles 47, 419–431.

[ref25] Khosravi A, Mohammadpoorasl A, Holakouie-Naieni K, Mahmoodi M, Pouyan AA, Mansournia MA (2016). Causal effect of self-esteem on cigarette smoking stages in adolescents: coarsened exact matching in a longitudinal study. Osong Public Health and Research Perspective 7, 341–345. 10.1016/j.phrp.2016.10.003 PMC5194218 28053837

[ref26] Knight Lapinski M, Rimal RN (2005). An explication of social norms. Communication Theory 15, 127–147.

[ref27] Lichter EL, Mccloskey LA (2004). The effects of childhood exposure to marital violence on adolescent gender-role beliefs and dating violence. Psychology of Women Quarterly 28, 344–357.

[ref28] Mackie G, Moneti F, Shakya H, Denny E (2015). What are Social Norms? How are They Measured? UNICEF and UCSD: New York.

[ref48] Marsh M, Purdin S, Navani S (2006). Addressing sexual violence in humanitarian emergencies. Glob Public Health 1, 133–146. doi:10.1080/17441690600652787 19153902 10.1080/17441690600652787

[ref29] Miller DT, Prentice DA (2016). Changing norms to change behavior. Annual Review of Psychology 67, 339–361. 10.1146/annurev-psych-010814-015013 26253542

[ref30] Morris MW, Hong Y-Y, Chiu C-Y, Liu Z (2015). Normology: integrating insights about social norms to understand cultural dynamics. Organizational Behavior and Human Decision Processes 129, 1–13.

[ref31] Rosenberg M (1965). Society and the Adolescent Self-Image. Princeton University Press: Princeton, NJ.

[ref32] Rosenberg M (1979). Conceiving the Self. Basic Books: New York.

[ref33] Schmitt DP, Allik J (2005). Simultaneous administration of the Rosenberg Self-Esteem Scale in 53 nations: exploring the universal and culture-specific features of global self-esteem. Journal of Personality and Social Psychology 89, 623–642. 16287423 10.1037/0022-3514.89.4.623

[ref34] Stark L, Asghar K, Meyer S, Yu G, Bakemore T, Poulton C, Falb K (2017a). The effect of gender norms on the association between violence and hope among girls in the Democratic Republic of the Congo. Global Mental Health 4, e1. 28596902 10.1017/gmh.2016.31 PMC5454793

[ref35] Stark L, Asghar K, Yu G, Bora C, Bavsa AA, Falb KL (2017b). Prevalence and associated risk factors of violence against conflict-affected female adolescents: a multi-country, cross-sectional study. Journal of Global Health 7, 010416. doi: 10.7189/jogh.07.010416. 28607672 10.7189/jogh.07.010416 PMC5460397

[ref36] Steinberg L (2005). Cognitive and affective development in adolescence. Trends in Cognitive Sciences 9, 69–74. doi: 10.1016/j.tics.2004.12.005. 15668099 10.1016/j.tics.2004.12.005

[ref37] Swallow SR, Kuiper NA (1988). Social comparison and negative self-evaluations: an application to depression. Clinical Psychology Review 8, 55–76.

[ref47] Tempany M (2009). What research tells us about the mental health and psychosocial wellbeing of Sudanese refugees: a literature review. Transcult Psychiatry 46, 300–315. doi:10.1177/1363461509105820 19541752 10.1177/1363461509105820

[ref38] Trott CD, Harman JJ, Kaufman MR (2016). Women's attitudes toward intimate partner violence in Ethiopia: the role of social norms in the interview context. Violence Against Women, 1016–1036. 10.1177/1077801216654018 27364004

[ref39] Trzesniewski KH, Donnellan MB, Moffitt TE, Robins RW, Poulton R, Caspi A (2006). Low self-esteem during adolescence predicts poor health, criminal behavior, and limited economic prospects during adulthood. Developmental Psychology 42, 381–390. 16569175 10.1037/0012-1649.42.2.381

[ref40] Viner RM, Ozer EM, Denny S, Marmot M, Resnick M, Fatusi A, Currie C (2012). Adolescence and the social determinants of health. The Lancet 379, 1641–1652. doi: 10.1016/S0140-6736(12)60149-4. 10.1016/S0140-6736(12)60149-4 22538179

[ref41] Uthman OA, Lawoko S, Moradi T (2009). Factors associated with attitudes towards intimate partner violence against women: a comparative analysis of 17 sub-Saharan countries. BMC International Health and Human Rights 9, 14. 19619299 10.1186/1472-698X-9-14 PMC2718859

[ref42] Young HP (2015). The evolution of social norms. Annual Review of Economics 7, 359–387.

[ref43] Zarrett N, Eccles J (2006). The passage to adulthood: challenges of late adolescence. New Directions for Student Leadership 2006, 13–28. 10.1002/yd.179 17225644

